# On Online Practices of Hospitality in Higher Education

**DOI:** 10.1007/s11217-021-09770-z

**Published:** 2021-06-28

**Authors:** Maria Grazia Imperiale, Alison Phipps, Giovanna Fassetta

**Affiliations:** School of Education, University of Glasgow, St. Andrews Building, 11 Eldon Street, Glasgow, G36NH UK

**Keywords:** Hospitality, Academic hospitality, Online, Affective, Derrida, Virtual pedagogy

## Abstract

This article contributes to conversations on hospitality in educational settings, with a focus on higher education and the online context. We integrate Derrida’s ethics of hospitality framework with a focus on practices of hospitality, including its affective and material, embodied dimension (Zembylas: Stud Philos Educ 39:37–50, 2019). This article offers empirical examples of practices of what we termed ‘virtual academic hospitality’: during a series of online collaborative and cross borders workshops with teachers of English based in the Gaza Strip (Palestine), we performed academic hospitality through virtual convivial rituals and the sharing of virtual gifts, which are illustrated here. We propose a revision of the concept of academic hospitality arguing that: firstly, academic hospitality is not limited to intellectual conversations; secondly, that the relationship between hospitality and mobility needs to be revised, since hospitality mediated by the technological medium can be performed, and technology may even stretch hospitality towards the unreachable ‘unconditional hospitality’ theorised by Derrida (Of hospitality: Anne Dufourmantelle invited Jacques Derrida to respond. Stanford University Press, Stanford, 2000); and thirdly, that indigenous epistemics, with their focus on the affective, may offer alternative understandings of conviviality within the academy. These points may contribute to the collective development of a new paradigmatic understanding of hospitality, one which integrates Western and indigenous traditions of hospitality, and which includes the online environment.

## Introduction

In education, the concept of hospitality has been theorised, unpacked and critiqued by different authors. The adoption of an ethic-of-hospitality framework that follows Derrida’s conceptualisations and the insoluble tensions he identified (Derrida 2000, 2001, 2002a, b) has been supported in particularly by Claudia Ruitenberg, who developed and applied such framework to curriculum and pedagogy (Ruitenberg 2011a, b, 2015, 2018). Other authors, writing on *Studies in Philosophy and Education*, have argued for a concept of hospitality that accounts for affectivities and the (spatial) materialities, developing the idea of *affective hospitality*, which would be the basis for transformational pedagogies (Zembylas 2019; Sinha 2018; Rasheed 2018; Bryzzheva 2018).

In higher education, the concept of ‘academic hospitality’ was developed in the early 2000s, by John Bennet, followed by a 2007 article by Alison Phipps and Ronald Barnett, admittedly written under very different technological, political and social conditions to those now prevailing. These authors note how ‘academic hospitality’ involves critical and celebratory conversations that occur in the academy, often during knowledge exchange events, for example at conferences, but also through reading, commenting, engaging with, and providing feedback on colleagues’ work. Despite its influence on the life of academics, however, the concept of academic hospitality has received scarce attention in the literature, and it has therefore not been revised in more recent times.

This article aims to participate in the conversations on hospitality in higher education. It offers new perspectives on the relation between mobility and hospitality, and it revisits the concept of academic hospitality in the light of changing circumstances and practices, in particular the huge increase in virtual connections that is a consequence of the covid-19 pandemic, but which had increased steadily since the work in 2007 and with technological possibilities. It does so by presenting and analysing empirical data taken from a research project which was conducted entirely online as participants were based in the Gaza Strip (Palestine), where the imposition of a blockade means that people have been living in a condition of forced immobility for well over a decade. The research project focussed on developing a series of participatory, online workshops to develop creative language teaching methodologies suitable for the context of protracted crisis of the Gaza Strip (Imperiale 2018). Conversations and relationships between the researcher and the participants were therefore forged entirely online. Relationships led to developing unintentional online practices of hospitality, which are discussed here.

In this article we explore whether it is possible to reconcile immobility and hospitality; what ‘virtual academic hospitality’ may look like; and whether an ethic-of-hospitality framework—grounded in Derrida’s aporias—can be integrated with Zembylas’ attention for affectivities and materialities and with indigenous epistemics on hospitality (George 2018). We attempt to answer these questions precisely in the spirit of academic hospitality. We do not aim to provide epistemological promises, but rather to continue and expand the conversations that this journal has hosted.

This article is structured as follows: in the first section we unpack and position ourselves within the debate on hospitality and education. In the second section, we present the concept of academic hospitality. In the third section, through the lenses of the ethic-as-hospitality framework, we illustrate and analyse empirical data and develop the concept of ‘virtual academic hospitality’.

## Hospitality and Education

Recent work published in this journal has opened up new trajectories for re-thinking hospitality in education as an affective, spatial, and relational practice (Zembylas 2019). Scholars have critiqued and taken further a Derridean hospitality-as-ethics framework, proposing a less metaphorical view of hospitality, and proposing one that accounts for emotions, spatiality and affectivity, encompassing everyday practices (Zembylas 2019; Sinha 2018; Rasheed 2018; Bryzzheva 2018). In this section we discuss our understanding of the current debate on hospitality in education, starting with Derrida’s work, presenting the critiques that have been moved to it, and finally positioning ourselves within the debate.

Jacques Derrida, along with Emmanuel Levinas, is likely the most influential intellectual on the concept of hospitality. Derrida’s work was seminal as he deconstructed and conceptualised the tensions, paradoxes, and contradictions of hospitality. Derrida himself wrote that ‘We do not know what hospitality is’, as it ‘rebels against any self-identity, or any consistent, stable, and objectifiable conceptual determination’ (Derrida 2000:6).

Derrida established the nexus between hospitality and ethics. In his own most cited words:

Hospitality is culture itself and not simply one ethic amongst others. Insofar as it has to do with the *ethos*, that is, the residence, one’s home, the familiar place of dwelling, inasmuch as it is a manner of being there, the manner in which we relate to ourselves and to others, to others as our own or as foreigners, ethics is hospitality, ethics is so thoroughly co-extensive with the experience of hospitality. (Derrida 2001, pp. 16–17)

Those words have been the object of several analysis and interpretations. Ruitenberg (2015) explained that Derrida’s hospitality is about the guest, and the position of the host is decentred: at the heart of this counterintuitive understanding of hospitality are not the rites of welcoming but rather the idea that the host is indebted to the guest (Ruitenberg 2015, 2011a, b). The three main aporias of Derrida’s construction and de-construction in hospitality, as pointed out by Ruitenberg (2015), are the tensions between invitation and visitation; protection and surrender of the host’s home; and reciprocity and unconditional giving. Unconditional hospitality (Derrida 2000) occurs without seeking to make the guest fit into the host’s space and therefore without affirming the host’s power over the guest; without being prepared to host; and without expecting anything in return.

In our understanding of Derrida’s writings, the idea of risk is the red thread that links together the aporias that Ruitenberg (2015) identified in Derrida’s work. The risk of allowing a stranger into the intimate domestic space, or even into a community’s public spaces, may result in a harmful encounter. However, it is precisely the embracing of this risk that distinguishes conditional hospitality (a welcome with limits) from an unconditional law of hospitality (Derrida 2000, 2001, 2002a, b). It is also why rituals of hospitality—of greeting, meeting and eating together are heavily codified across cultures and societies. Unconditional hospitality represents the possibility to make the impossible work, and to give up control.

In one of his perhaps *less* cited passages, Derrida wrote:

To be hospitable is to let oneself be overtaken [surprendre], to be ready to not be ready, if such is possible, to let oneself be overtaken, to not even let oneself be overtaken, to be surprised, in a fashion almost violent, violated and raped [violée], stolen [voleé] [...], precisely where one is not ready to receive—and not only not yet ready but not ready, unprepared in a mode that is not even that of the “not yet”. (2002a: 361)

Our understanding of Derrida’s unconditional hospitality is encapsulated in this sentence: ‘to be ready to not be ready’, to give up control almost in a violent way, radically, in order to be open so that someone can come in: this is the paradox of the impossibility of hospitality. It is perhaps worth adding that in feminist psychoanalysis, notably in the writings of Irigary (2013), the factor of consent in hospitality is key, as she explicates in her re-figuring of the annunciation and the consent to a ‘hospitality’ of the womb. The online environment, even though it requires planning, is also highly unpredictable and requires a letting go of control, at least to some extent, as will be further explained in the following sections. The notions of ‘giving up control’ and ‘taking risks’ are crucial to our work with partners in the Gaza Strip, and will guide our argument throughout this article.

For unconditional and radical hospitality to exist, notes Derrida (1998), I must not expect nor invite the other. The other could come to me unexpectedly, at any time. They may be friend or foe, and, without knowing which, I must be open to their visitation. Being hospitable in this unconditional way is—essentially—stretching. However, the possibility to *stretch towards* this impossibility, as best as we can, is still open to us, though it will leave its marks. Derrida’s unsolvable tensions between invitation and visitation, protecting and relinquishing, reciprocity and unconditional giving do not lead to paralysis, but rather to a willingness to push the threshold of what is possible. His work challenges us to explore a sort of when-is-enough-enough conundrum: how do we establish where the threshold between what is possible and what is impossible lies? This idea should stimulate us in leaning/stretching towards the impossible. We will return to the idea of ‘stretching towards’ in the following section.

Derrida’s work has been also critiqued. For example, Bulley (2015) explains that while Derrida presented strong statements such as ‘hospitality is culture’, ‘ethics is hospitality’ and that before that hospitality ‘is the principle of ethics’ (Derrida 2001)—those statements have not been further unpacked, referring in particular to the spatial dimension of hospitality. In addition, Bulley (2015) argues that Derrida’s understanding of ethics and of hospitality are linked to sovereignty, to state and macro-politics (e.g. his activism in favour of the *sans-papiers*), and this perhaps has obfuscated the analysis of everyday practices of hospitality (Bulley 2015). Starting from this critique, Bulley suggests to move beyond Derrida: he argues, convincingly, that hospitality is spatial, relational and has an affective dimension. Spatial, as it involves some sort of borders, *physical* borders, to be crossed. Affective and relational, as there is a distinction between a charitable act and a hospitable act, between acting responsibly for the common good and being hospitable. According to Bulley (2015), and we would concur on this point too, what makes the difference between social responsibility and hospitality is the presence of affectivity that characterises the latter.

In the field of education, Claudia Ruitenberg (2015) worked through Derrida’s framework, and proposed a hospitality-as-ethics framework for and within education. Acknowledging the importance of immanence, she explains that “hospitality in education can only be invented anew each time in a *particular* context, with a *particular* host and a *particular* guest” (ibid., p. 70, italics in the original). Analysing Derrida’s definition of hospitality—with reference to the quote we cited above in which Derrida links together ethics, culture and hospitality—she suggests that hospitality is caught between unconditional hospitality and attending to the material and to the daily practices which unfold in a particular moment and in a particular place: she points out that Derrida himself (2001, pp. 16–17) explains that hospitality has to do with residence, with places, with dwelling, merging the theory–practice divide, and acknowledging the importance of the spatial and immanent dimension of hospitality.

Importantly, Ruitenberg (2015) applies this framework to develop a hospitable curriculum, and to illustrate what a hospitable pedagogy looks like. She retains the tensions between ‘the law of hospitality’ and ‘laws of hospitality’—that is, between ethics and politics—by offering a vocabulary and ideas to apply the ethics of hospitality in the context of the classroom. An ethics of hospitality serves the purpose of developing hospitable educational contexts, or at least of protesting the contextual conditions that hinder hospitable practices (Ruitenberg 2015, 2018). This can be particularly challenging since, as Doris Santoro (2016) pointed out, new teachers are encouraged to be hospitable towards their students precisely as they enter a profession where they do not receive hospitality, since the value of the teaching profession is often unrecognised, conditions are far from optimal, and teachers’ work is, in many cases, inadequately remunerated.

Critiques based on the metaphorical enactment of hospitality have been moved both to Ruitenberg’s framework and to Derrida (Zembylas 2019; Santoro 2016; Eppert 2011; Sinha and Rasheed 2018; Sinha 2018; Bryzzheva 2018; Rasheed 2018). Building on Bulley’s idea of hospitality as spatial and affective (Bulley 2015), Zembylas (2019) further develops the concept of *affective* hospitality in education by drawing on affect theory. He argues for a re-conceptualization of hospitality as a material and affective practice which needs to pay attention to how bodies interact in particular places, building or demolishing ‘atmospheric walls’ (Ahmed 2014). Shilpi Sinha (2018), Shaireen Rasheed (2018), and Lyudmila Bryzzheva (2018) in their respective essays, similarly discuss ‘racialised bodies’—referring to non-white bodies—pointing out that these have received scarce attention in the hospitality-as-ethics framework. The authors’ dissatisfaction with such framework alludes to the impossibility to make it work in the classroom where there are non-white bodies and spaces: racialised students and teachers, inhabiting spaces marked by ‘whiteness’ as these are supplanted by white structures and white social systems and order, are excluded from a truly hospitable framework.

Ruitenberg (2018) responds to these claims by acknowledging and interrogating the spaces in which she works and considering her position and privileges as a white academic. As a result, she suggests that it is not necessary to reject Derrida’s hospitality-as-ethics framework. On the contrary, adopting hospitality as an ethical framework allows a critique of educational contexts and racialised spaces which do not encourage neither favour the practice(s) of hospitality (Ruitenberg 2018). Hospitality-as-ethics is therefore not metaphorical, but it is material and, in the context of education, has the potential to open up transformational spaces and pedagogies.

A focus on affectivity and spatiality is found in indigenous epistemics (Santos 2018), for instance in the Māori’s term for hospitality, *manaakitanga.* As the Māori dictionary describes it, *manaakitanga* is “the process of showing respect, generosity and care for others’ (Māori dictionary, online), and this is significant for our discussion. Constituents of the word include the verb ‘*manaaki*’ which means “to support, take care of, give hospitality to, protect, look out for, show respect, generosity and care for others” (Māori dictionary, online). These values do not precisely overlap with the Oxford English Dictionary definition of hospitality as “the act or practice of being hospitable; the reception and entertainment of guests, visitors, or strangers, with liberality and goodwill” (OED). The latter definition refers to a ‘hospitality industry’ which has subsumed much of the academic hospitality discussed by Phipps and Barnett (2007). The definition of the Māori terms, on the other hand, retains a focus on performativity and ceremonial wrapping of *care* as an ethic akin to Derrida’s invitation to stretch towards unconditional and aneconomic hospitality.

Derrida himself experienced Māori hospitality through the ritual of a *Pōwhiri* during his visit to Aotearoa New Zealand, while his conceptualisation of hospitality was developing (George 2018). A *Pōwhiri* is a powerful, ceremonial, ritualised meeting, a form of international relations whereby through a series of protocols that stress people’s differences, visitors are turned into members of the *marae* (George 2018)*. Marae* indicates at the same time both a physical domain and a place of meeting for social and sacred activities for tribe and sub-tribe. As a meeting place and place of hospitality it is also highly codified with *tikanga*—protocols. George, an indigenous anthropologist, describes the ritual as “moving people through space’, a space ‘where relationships are assessed and negotiated. It can be a space of creation, of innovation, as well as connection” (George 2018:9). Linda Tuhiwai Smith (2012) captured a picture of that moment and, during a lecture explained that the picture was important as it represented the *space* between the guest (Derrida) and the group representing Auckland University (George 2018).

We can finally bring together Ruitenberg’s and Derrida’s analysis and understanding of a hospitality-as-ethic framework in education, Zembylas’s (2019) work on the importance of affectivities and materialities of hospitality—for hospitality is inherently relational—and a focus on care and on materiality as it emerges in the Māori’s definition of hospitality. We argue that precisely by attending to the everyday, material and visceral practices of hospitality and care within educational settings and by analysing and foregrounding these, it is possible to transform places, relationships, pedagogies. By engaging in practices of hospitality—of *manaakitanga—*in educational settings, rather than perceiving hospitality just as a metaphorical representation, we can also stretch towards unconditional hospitality. Manaaki, it should also be noted, is a compound word, made up importantly of the untranslatable term *mana*—the power of elemental forces as embodied in an object or person, and also of prestige and moral authority. *Aki* means encouragement and so emboed in the values of *manaaki*, of what in English is translated as hospitality, are quite different but revealing notions—that the practice is a duty and encourage which will enhance the prestige and standing in moral authority of those who engage in it well.

These theories complement our understandings of hospitality in terms of emphasising the importance of care, and of embodied and visceral practices; and without these, our understanding of hospitality, especially when working in Global South^1^ contexts—as with our work in the Gaza Strip—would remain partial (Buissink et al. 2017; Smith 2012). These concepts are relevant to our discussion of ‘virtual hospitality’ as they help us understand how we lived and performed care and hospitality with our colleagues in the Gaza Strip. Our work in Gaza has enabled a first deconstruction of many of the assumptions in earlier work in academic hospitality, and of what the stretch towards unconditional hospitality might require us to learn, to decolonise and to decreate (Phipps 2019).

## Academic Hospitality

The debate on hospitality in higher education has been initiated by John Bennett in the early 2000 (Bennett 2000, 2003). Bennett opened his article (2000: 23) with the following statement, which is worth citing in full:

A key virtue for the academy is hospitality—the extension of self in order to welcome the other by sharing and receiving intellectual resources and insights. […] Admittedly, my view is not a common one. Hospitality is often taken to mean a bland congeniality. As theologian Henri Nouwen notes, for many if not most of us, hospitality suggests ‘tea parties, bland conversations, and a general atmosphere of coziness.

Bennett (2000: 28) developed his view of intellectual hospitality as achieved in conversation: being engaged in ‘honest, critical dialogue whose goal is to clarify meaning, not to win or to establish superiority’ means to practice intellectual hospitality. He differentiated two levels of conversations: the first consists of offering and receiving (i.e. to exchange knowledge providing insights into one’s *own* position), and the second consists of offering feedback on the *other’s* position. Only when the first level has been practiced sufficiently, then it is advisable to move onto the second level. He highlighted that in academia, hospitality goes beyond being ‘courteous and civil’ but being academically hospitable rather means ‘working toward mutual interaction and reciprocity’ (Bennett 2000: 25). Considering the three domains in which the academy operates—teaching, scholarship and service—Bennett argued for changes in management, condemning ‘insistent individualism’ and suggesting standards to develop a healthy, collegial learning community.

Phipps and Barnett (2007) also worked on the concept of ‘academic hospitality’. Building on Bennett’s ideas of intellectual hospitality described above (i.e. offering, sharing, providing and receiving) and working through Derrida’s writings and Paul Ricoeur’s linguistic hospitality (Ricoeur 2004), Phipps and Barnett noted that ‘academic hospitality, as a practice, [was] under threat’ (p. 327), referring to both the flow of knowledge and the lack of good practice and collegiality that Bennett(2000) highlighted. In some ways their work was prescient, predicting the practices of the ‘Hostile environment’ of the UK Home Office, and the many visa refusals for Palestinian partners which were experienced during the project discussed in this article and many other projects besides. What the authors failed to acknowledge at the time was the extent to which the impossibility to practice academic hospitality in person, the denial of the right by states, would lead to a creative resistance through new online practices.

Phipps and Barnett (2007) described four forms of hospitality: the material form, the epistemological form, the linguistic form, the touristic form, all of these being dependent upon travelling. Within these forms, academic hospitality is enacted in *celebratory*, *communicative* ad *critical* modalities. They warned against adopting just one mode of hospitality, since that would mean a simplification of the tensions and paradoxes of both hospitality and academic life. They also mentioned the importance of technology and of virtual materiality, as “colleagues are met, articles are consulted and conferences are held via the Internet” (p. 240), adding that a practice of virtual hospitality holds its very own challenges and questions. However, at the time of writing, the virtual was mainly experienced through growing numbers of emails and the beginning of the hegemony of the ‘website’ in academic life, which were nascent and still suffered from technological problems. Consequently, the materialities of virtual hospitality were not further unpacked.

Phipps and Barnett (2007: 253) concluded by noting that academic hospitality:

opens us out into a messy, committed business that seeks to work out, practice, and come to learn through celebration, communication and criticality within the possibilities of academic life: the concept helps us conceptualize a metaphorical and also a performative academic life. […] The art of conversation is the art of hospitality’.

Conversations are at the same time both metaphors for the intellectual engagement and openness that constitute academic hospitality, and an enactment of practices of hospitality. Conversations allow hospitality to be performed.

Our critique of academic hospitality in term of intellectual conversations focuses on three main points: firstly, understanding (academic) hospitality in terms of intellectual conversations seems to suggest that the tensions and complexities of hospitality in higher education are reduced to communication related to the occupational domain; secondly, the relation between (academic) hospitality and travelling/crossing borders may need to be reconsidered, for many practices of hospitality, nowadays, do not (cannot and, considering environmental consequence, also should not) involve travelling; thirdly, as mentioned in the previous section, the focus on conversation fails to acknowledge the dimensions of practice, ceremony and care of indigenous epistemologies (the *manaakitanga*) and their importance in both online and face to face experiences of academic hospitality, in particular in interactions with and between the Global South and Global North.

In order to attend to the first point, we turn now to the anthropologist Tim Ingold. Although Ingold has never mentioned hospitality in his writings, he recently penned a brief and compelling book entitled *Anthropology and/as Education* (2018) in which, drawing on the writings of John Dewey, he argues against the idea that education is the transmission of knowledge, and posits that it is rather ‘the practice of attention’. The word ‘attention’ is derived from Latin *attendere*, and it means ‘to stretch’ (*tendere*) + ‘towards’ (*ad*), to lean towards, to reach out towards. This is what Ingold refers to as the ‘stretch of life’. Education allows us to stretch towards life. *Attending* as the stretch of life has some related meanings: firstly, actively listening (as we reach out towards a distant sound); secondly, waiting; thirdly, being present; fourthly, going along. Ingold adds to the ‘stretch of life’ another meaning, that of ‘longing’. Longing is the added dimension of temporality—as life ‘is not merely lived in the here and now but it is stretched by a memory of the future that itself allows every present moment to be a new beginning’ (Ingold 2018: 45). This work on ‘leaning towards’ is also elaborated from a feminist perspective by Adrianna Cavarero in her book *Inclinations: A Critique of Rectitude* (Cavarero 2016) in her substitution of the notion of homo erectus as the dominant posture for civil life, with that of inclination, of leaning towards the other in an attitude of care. Her critique allows for a feminist and womanist intersection with concepts of care in indigenous conceptualisations of hospitality.

The etymology of the word ‘education’, notes Ingold (2018), again comes from Latin. It is derived from *educare* (*e* + *ducere*) where the prefix *e* comes from *ex* which means ‘out’: the meaning of education is therefore to ‘lead out’. The prefix ‘*e’* is fundamental here—the meaning of education and, implicitly, the task of education, ought to be to lead someone *out*, and not to instil knowledge *in* someone. Education is an invitation to lead out,

to break out of the security of our defensive positions, take our armour, and meet the world with open arms. It is a practice of disarmament. […] it is exposure rather than immunity; it renders us vulnerable rather than powerful, but by the same token, it values truth and wisdom over knowledge.’ (Ingold 2018:67)

Education is risky, it makes us vulnerable as we open to the other, it is weak rather than strong. It is done with attention, with care. We can see, in Ingold’s view of education, parallels with the discussion on hospitality we developed in the previous section, where hospitality is unreserved openness to be surprised and overtaken (Derrida 2002a, b), a willingness to be vulnerable.

Our reading of Ingold’s vision of (higher) education also alludes to the practices that Bennett (2000) first, and then Phipps and Barnett (2007), have termed ‘intellectual hospitality’ as one of the components of academic hospitality: open, critical, celebratory, responsible conversations that aim at leading out. While, as noticed earlier, Ingold never refers to these practices in terms of hospitality, he postulates what Higher Education ought to be and to do, aiming for the openness, risk-taking and vulnerability that are at the core of unconditional hospitality. This seems to imply that what Bennett (2000) understood as ‘intellectual hospitality’ would be, in Ingold’s view, approaches that constitute of the essence of higher education. What is it, then, that is specific to academic hospitality and is not just ‘good academic practices’?

In order to attend to the second conundrum mentioned above, that is, the relation between hospitality and mobility, and to the specificity of ‘academic hospitality’ we present empirical data that we analysed in terms of ‘virtual academic hospitality’. These data seem to encompass both conundrums: the practices of hospitality presented here cannot be analysed in terms of ‘conversations’ only, and they also show that ‘hospitality’ is not always dependent upon travelling. In addition, these can be considered at the intersection between an ethic-of-hospitality framework in education, while also attending to affectivities and materialities (Zembylas 2019), and dimensions of care, embodiment and performativity (Santos 2018), such as those inscribed in *manaakitanga.*

## Virtual Academic Hospitality

The study on which this paper is based was a participatory action research with pre-service English teachers based in the Gaza Strip (Palestine). The Gaza Strip is a small piece of land with a highly dense population: 2 million Palestinians live in a ribbon of 40 km in length and between 14 and 16 km in width. The Gaza Strip is under blockade since 2007, when Israel declared it ‘hostile territory’. Unemployment rate, poverty rate, lack of appropriate infrastructures, frequent and extensive power-cuts, three military operations carried out in 2008, 2012, and 2014 make the Strip an ‘unliveable place’ (Fassetta et al. 2020). The blockade has worsened the living conditions of people in the Strip, leaving a profound impact on people mental and physical wellbeing, and limiting peoples’ freedoms and capabilities (Smith 2015).

As a consequence of the blockade, movements in and out of the Strip are virtually impossible: this is the reason why most international research projects have to be carried out online, navigating all the challenges that come with this form of collaboration. A strong determination to make connections happen is at the heart of any type of research conducted in these conditions (Fassetta et al. 2020; Imperiale et al. 2017).

Engagement with the Gaza strip came through the work of Keith Hammond (Hammond 2012) and his leadership of the European TEMPUS funded project Life Long Learning in Palestine, for which Phipps was Co-I. Phipps travelled to the Gaza Strip and experienced academic hospitality in the Strip in 2011 (Phipps 2014). Since then, despite over ten years of funding which would enable a return, none of the authors of this article have been able to access the Strip: the ongoing siege, and then in 2014, the military operation launched against the Gaza Strip, followed by a strict closure of borders, and the impossibility of securing either visas or insurance or travel permissions from our institution, meant that all our research projects in cooperation with the Gaza Strip had to move online, and with it the majority of experiences of academic hospitality.

The data we draw upon were part of a project on exploring and developing localised, critical and creative English language teaching methodologies (Imperiale 2018, 2021). The research study consisted of a series of participatory workshops during which thirteen pre-service English teachers worked entirely online, in collaboration with Imperiale, to co-create and explore the potential of creative pedagogies. The overarching aim was to ultimately develop a language pedagogy that would work towards wellbeing, resilience, and the development of students’ voice and agency through a foreign language (Imperiale 2018). How relationships between the researcher and the participants were established, working within the challenges of the online environment, has been the focus of another paper (Imperiale, in press) in which Imperiale explores how, in order to unpack the development of those relationships, she had to attend to materialities and affectivities. Relationships were shaped by the network, and in return, the researcher and pre-service teachers acted upon the materialities of the network. Dynamics of power were also altered thanks to the materialities of being online: participants had to physically mute Imperiale on Skype, they had to move her ‘body-on-the-screen’ in order to facilitate her capacity to see around the room in which participants were located. This rested on a disposition, as Author 1 allowed herself to take the risk, to make herself vulnerable and open to the other (Ingold 2018) by situating herself in the encounter as someone who could be ‘muted’ or ‘carried around the room’ (Imperiale 2018). These instances, in addition, far from being metaphorical, deeply changed, and strengthened, the relationships between the researcher and the pre-service teachers.

The ‘research encounters’—and relationships—culminated in moments of shared hospitality—or better—of ‘virtual (academic) hospitality’. Moved by a strong willingness to meet each other, to host each other, to show each others’ dwellings, the researcher and the participants relied upon the rituals they all were familiar with, that of commensality and of the exchange of gifts, that were translated into online practices.

### Virtual Commensality

Being in an online environment, one of the limits imposed by the physical distance and the virtual materiality, is the impossibility to share real moments of commensality. According to Boudou (2012), commensality is equivalent to signing the pact of hospitality since it is the public confirmation of welcoming, and in this sense, it embodies and phenomenalizes hospitality. Derrida (2000), conversely, argues that hospitality based on invitation is ‘welcoming with limits’ since it is enacted upon preparation. Only when you are ready not to be ready, does hospitality become ontological and epistemological ethics. However, being ready not to be ready requires some preparation and it is a process that comes with time and with experience: it is perhaps the disposition to constantly stretch towards the world.

Relying on the familiar practices of hospitality through commensality, during the last day of the workshop series, the trainees brought a cake to celebrate the end of the workshop series. That was an intense moment, in which the will to be hospitable and to share celebratory food faced the virtual-material reality and the impossibility and limits of virtual hospitality.

The frustration caused by the impossibility of sharing commensality was mitigated by the happiness of sharing a cup of tea on Skype and by seeing the enthusiasm and gratefulness of having embarked together along this research—and educational—journey.

Cake time. F. slices the chocolate cake she made. I am overwhelmed by happiness in seeing the joy they [the participants] spread. Music in the background. N. invites me to get a cup of tea, so at least I can share a tea with them. I put my pen down, it is not the time to take notes anymore. I want just to be filled with their laughter and my tea. Everybody is smiling. Tears are coming soon. (Author 1, research journal)

**Fig. 1:**
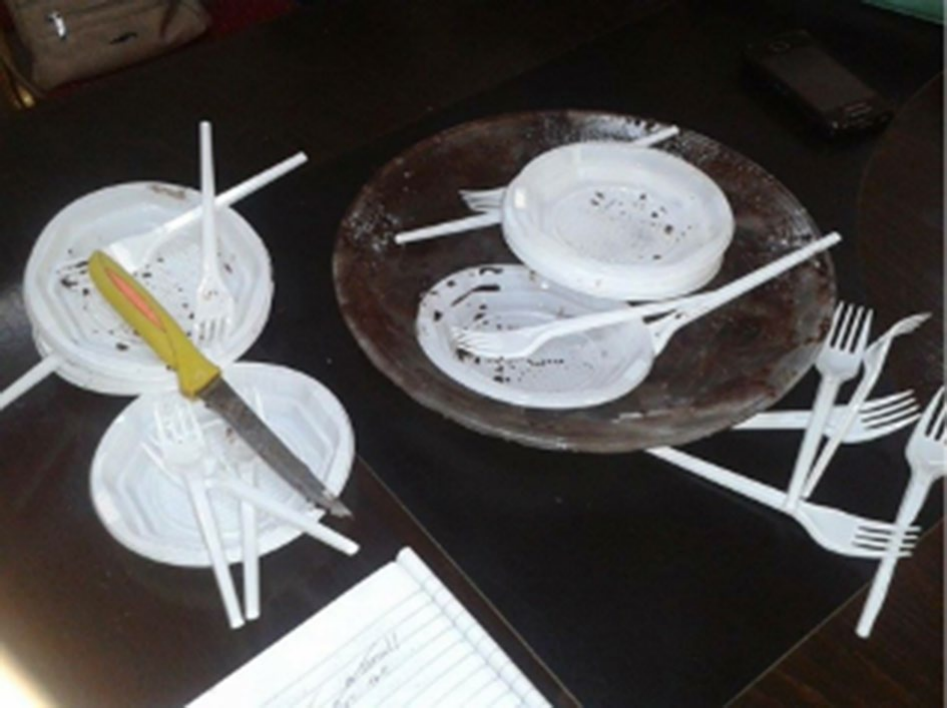
Empty plates during the last workshop

Similarly, several trainees described that moment, in their evaluation sheets, as one of the happiest moments shared during the course. A few days later, some of the young women shared the pictures they took during the course. It was only when the researcher received the following picture via email that her sense of bitter-sweetness and frustration became more acute (Fig. 1):

Skype made it real, you were with us... but it was a pity that you could not eat the cake with us... much love to you and I will invite you to my home when you will be in Gaza and will cook for you too much food. <3

(A., personal email)

The researcher’s response in her research journal shows her frustration at the impossibility to cross borders:

A. sent me the pictures of the empty plates on which they ate the cake. In that celebratory moment, the impossibility of travelling and crossing borders made itself heard quite firmly. We all laughed then, shared a cup of tea, enjoyed the music that the girls played at their end. Despite the happiness, I wasn’t where I was supposed to be though. I should have been with them, laughing, dancing, and sharing food. But somehow, we managed to share a cup of tea, on Skype. But was that enough? (Author 1, research journal, 15 May 2015)

After the workshop series, when conducting follow-up interviews with the trainees, the ‘Skype-cup-of-tea’ became a ritual of the established online community, and was a source of recurrent conversations similar to this one:

Author 1: How is your tea? [laughing]

A: You put mint?

Author 1: Yeah, fresh mint!

A: And how many spoons of sugar? […] (Interview with A.)

The impossibility of practicing hospitality with all its paradoxes (Derrida 2000) emerges in virtual academic hospitality. Despite the fact that the trainees and the researcher did not share the same food and were not in physical proximity, the will to be hospitable, to share and to be integrated made everyone involved shape familiar practices of hospitability, and transformed what seemed to be impossible into an unusual ritual of ‘virtual commensality’ (Imperiale 2018), a ritual which has since become much more common in recent times, as friends, family or colleagues separated by the Covid-19 pandemic—and consequent lockdown—have taken up similar forms of online commensality.

The virtual ritual of the ‘Skype-cup-of-tea’, with its attempt to share the same substance and the same space, stood on one hand as the possibility to open oneself to the culinary habits of the other, and on the other hand it was sometimes a restful moment in the midst of poignant and difficult conversations. During the follow-up individual interviews, it also involved accessing the intimacy of each other’s kitchens, simultaneously, and meeting respective family members. Some of the participants during the follow-up interviews met the researcher’s family members, and accessed her kitchen and living room, as she did with theirs. They reciprocally performed greetings in Arabic (participants’ native tongue) and Italian (the researcher’s native tongue), and translated at times when family members were present, enacting linguistic hospitality (Ricoeur 2004). Participants were keen to introduce the researcher to their families, and a large part of the Skype interviews consisted of sharing moments together and visiting each other’s’ houses.

In virtual hospitality, the ontological risk of the unknown Derrida (2000) talks about does not encompass the risk of violence and physical harm (although harassment, intimidation and bullying are a possibility) from the ‘other’. Rather, as happened during the research project discussed here, the risk of the unknown manifested in the frustration of experiencing the limits of the medium. Both the participants and the researcher wished to escape the constraints of Skype and to be able to visit each other’s homes in all their physicality. Experiencing the limits of immobility involved a certain degree of distress and frustration. Virtual hospitality can also hurt.

These online practices have since become much more common during the Covid-19 pandemic: it is now frequent to have online work meetings, during which someone’s children might join conversation, or to inquire about what is happening within the intimacy of the space of the house, which would not usually be open to colleagues. We acknowledge that there is now the possibility to have virtual backgrounds, to turn cameras on and off as required, but the work interaction still invades the intimacy of the home. This conflation of once separate spaces may be a form of hospitality, where someone enters a space even if not invited, and where others are willing to let you in, blurring the boundary between host/guest. Our co-workers are ‘crossing the threshold’ of our houses, even if they are not invited, during online meetings. Even though we decide to use a virtual background, and they are therefore not able to see and to fit into our domestic space, still their presence may be felt and perceived by our family members. The spatiality of the house may have to change, as partners and children need to let family members work. Those we share our homes with perform hospitality by sacrificing their own space to allow our colleagues in. For those living in small accommodation, and for those who have young children, this can be very challenging, and cause conflict and frustration. Virtual hospitality can also hurt.

### Online Gifts

Similarly to the ‘impossibility of hospitality’ and as part of hospitality itself, Derrida discusses the ‘impossibility of the gift’ (Derrida 1992: 7). He states that a gift must be ‘aneconomic’, meaning that it should be out of the economic circle for it to be a genuine gift: it should overcome the logic of giving and receiving upon calculation.

Not that it remains foreign to the circle, but it must keep a relation of foreignness to the circle, a relation without relation of familiar foreignness. It is perhaps in this sense that the gift is the impossible. (Derrida 1992: 7)

In this sense, the gift should not be measured, neither should it be subject to prior calculation: the gift should transgress its borders, being an event of madness and immeasurable excess. Critiquing the work of Mauss (1954), who explains the economy and reciprocity of the circle of the gift and the counter-gift, Derrida (1992: 14) states that ‘at the limit, the gift as gift ought not to appear as gift: either to the donee or to the donor’. As with hospitality, he does not believe that impossibility leads to paralysis, but rather opens up possibilities for practicing unconditional gratefulness. It also fits with the enhancement of prestige discussed earlier when excavating the protocols of hospitality underlying Māori understandings. These gifts reflect the moral recognition as opposed to any material need for the guest to receive food or shelter, or entertainment, given the virtual distance and proximities in play.

After the workshop series the trainees sent the researcher ‘gifts’ virtually: gifts were aneconomic, did not have monetary value but rather carried profound symbolism. They gifted pictures, incomplete poetic written texts, and a combination of the two. The researcher reciprocated with pictures of her homes, both of the one where she grew up in Italy, and other places in which she felt at home in the UK. She also sent them pictures of green and humid landscapes over the summer, hoping to convey a sense of relief from the heat of the Gaza Strip. We discuss here some of those digital gifts.

#### The Gift of a Poem

During the workshop series participants composed numerous poems, some of which were written for the purpose of the workshops. Other poems, more personal ones, arrived unexpectedly via email:

**A Farewell to Grazia**

To remember that we will not be to this place anymore

oh, no, it is really so sore

forgive me if I tell you that we will miss you dear

since the real feelings I didn't reveal

make sure that we will not miss you

but the fact is that our heart will be forever with you

Today is not to say Good bye

Today is not to shed tears or cry

Today is to leave you with a smile

when the watch six pm strikes

all will leave

and none you will find

but our heart will be waiting there,

listening to the echos of our voices

and laughters stored in our minds

[A.W.]

In the shared highly languaged space of the workshops, words were the most precious gifts. When borders cannot be crossed, language and images can cross borders and the limits of physical immobility. Words did not only accompany the gift—but were the gift itself. The researcher was gifted with poetic language. The gift, as Derrida wrote (1992), arrived in narratives. The poem received via email, was accompanied by a caption that encompassed the ontological nexus between giving-being. The trainee acknowledged her ontological becoming during the workshop series, and her ‘new’ being found a nexus with her giving:

In this course I discovered that I am a poet. During this course I wrote 11 poems! I always liked poems but I didn’t know I could write so many! Thank you dear. This poem is a gift for you, it is not completed but Insha Allah I will finish it to be splendid. [A.W., personal email]

The legacy of the yet-to-be-completed text seems to be the legacy of expressing the inexpressible feelings of A.W.’s ontological process of becoming. The ‘incompleteness’ of the yet-to-come gift seems to be a rupture in the circle of gift and counter-gift, of calculation and expectation. It announces its potential development, without putting the recipient in the position of owing, of holding a debt. The gift, in the moment when it was received, at the same time appeared to be both a present and an incomplete-present. The trainee, sending an incomplete poem, made the recipient wait for something.

Waiting meant, as hearts were at the end of the course, being suspended between the memories of our past laughter, the yet-to-be future, and the present. This gift—as time—escaped the moment in which it is given. Derrida (1992) writes that a gift gives and demands time. The present of a present is imperceptible as it ceases to be a gift in the moment it is perceived as such. The incompleteness of this gift, on the other hand, makes the impossibility of a radical gift, possible.

#### The Gift of a Photograph

**Fig. 2:**
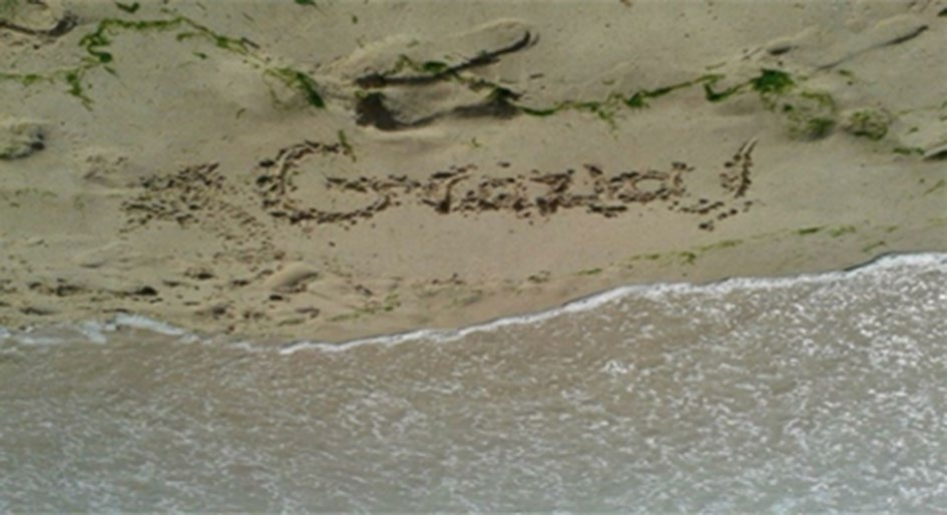
Picture of the Gaza beach as a gift from participants

**Fig. 3:**
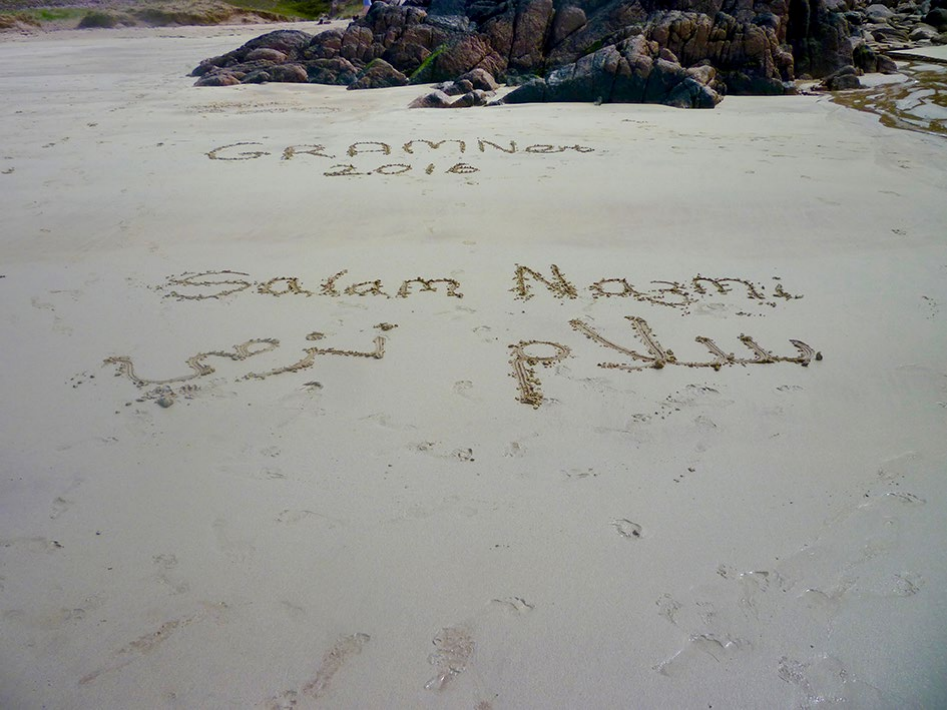
Picture of the Scottish beach as a gift to participants

The trainees also sent pictures of Gaza (Fig. 2), in particular of the Gaza beach and the Mediterranean Sea. The beach captured in the pictures was never untouched, rather participants left their prints on the seashore. Close to the sea, they took pictures in the liminal moments when the waves have not washed the words away, yet. The researcher received pictures with her name, their names next to it, with the map of Palestine drawn on the seashore. Similarly, we sent them pictures of our Scottish beaches, where we drew our partners’ names and multilingual greetings, in English and in Arabic (Fig. 3).

The relationship between (home)land and language is quite evident. Language is imprinted, precariously, on the soil. The message gets across the Mediterranean through the online medium and brings the researcher virtually there. Her name printed on the land in which the participants dwell, symbolizes her presence on their land. It symbolizes the impossibility of welcoming her on their beach. Those pictures and the precarity they captured on the shore evoke a sense of radical hospitality—with its characteristic temporality, its unconditional ethics and aesthetics. Photographing that precarity makes the moment eternal and shareable, and possibly stretches it out to the future. What is radical about this gift, we believe, is the creative form through which the impossibility of hospitality is performed. The picture with the name of another person on the soil symbolizes the impossibility of mobility—the impossibility to cross borders—and the stretch towards making others present on our land, in our soil. We interpret this as a form of radical hospitality that relates to Derrida’s (2000) aporias of invitation and visitation: others are inviting us and, as we are unable to accept the invitation, they bring us there by writing our names where they would like us to be. The inscriptions equally demonstrate ‘mana’—that the person whose name is written on a board, or piece of paper or in the sand is being thought of or remembered despite there being no necessity for this. It is a gratuitous act of giving, though, as Mauss (1954) states, the indebtedness created by these actions will orchestrate its own moral and affective economies.

What we have presented above as virtual commensality and online gifts, we believe, challenge the presuppositions that hospitality requires physical proximity and travelling: the other can enter through different channels, even though, admittedly, there is something irremediably lacking when we do not share the same space, as also reflected in the fieldnotes presented above, ‘But was that enough?’. Perhaps, what is lacking is the visceral experience of spatial proximity.

On the other hand, this also confirms that hospitality is dependent upon the will of meeting the Other in language, dependent upon the will to establish human connections. Our online relationships, and hence virtual academic hospitality, were possible because of our willingness to establish human connections, in spite of the fragmentation and interruptions of our network but also thanks to the shared effort to (re)connect this entailed. Materiality and relationality intersected in the network and this at times made us feel frustrated, and in other moments carried an unexpected and overwhelming fulfilment complemented by Skype-cups-of-tea, pictures, joy and unconditional sharing beyond the threshold of the screen:

Hospitality can only take place beyond hospitality, in deciding to let it come, overcoming the hospitality that paralyzes itself on the threshold which it is. It is perhaps in this sense that ‘we do not know what hospitality is,’ and what hospitality awaits. (Derrida 2000: 14)

In the online context in which this research study was conducted, we had to rest upon critical and celebratory conversations that always accompanied our will to *relate* to ourselves and to others, to be open to the unfamiliar, unknown context of the online network that we found ourselves connected by, and that we shaped and in turn were shaped by. It was precisely in conversations—and within its opposite, i.e. thanks to the interruptions—that we manifested our will to be hospitable, our will to overcome the obstacles imposed by the virtual reality.

However, importantly, it seems to us that the conversations that allowed practices of academic hospitality were the ones that were not related to work, those that were not ‘intellectual conversations’, neither ‘academic conversations’, those that did not involve providing feedback on each other’s work; were rather the ones about our respective families, our struggles, our joys, our respective vulnerabilities, our different teas, and our gifts. Those were the moments that resulted out of affectivity and of attention, rather than reasoning on specific research-related issues; those were the ones that allowed us to *stretch towards* each other, involving not only academic conversations, but other practices of conviviality.

## Conclusions

To learn which questions are unanswerable, and *not to answer them*: this skill is most needful in times of stress and darkness.

Ursula K. Le Guin (1969: 110 emphasis in the original)

This paper contributes to the conversations that this journal has already hosted on hospitality, in particular those on hospitality in educational settings. It is an attempt that ultimately aims to reach out to other colleagues and scholars whose work in this area is already well-established and that opens up a set of questions and conundrums, in the realisation that—in the contradiction and uncertainty created by ontological and epistemological shifts—the asking of the question may be, at least for now, the only possible answer.

Hospitality is one of the tenets of our age, based on mobility, movement, infinite possibilities to cross borders. As a result of the covid-19 pandemic, conferences have been cancelled, teaching has been moved onto online platforms, physical and social distancing are the buzzwords. Most academics have, more or less willingly or confidently, moved to create academic communities online, experiencing the limits and freedoms of this mode of working. Within this context, this article hopes to contribute to a growing collective reflection on what online academic hospitality can be and do, but also leave open the question of what is lost, or altered.

We argue that the opening of territories and homes unconditionally and unreservedly, as Derrida argued, is a fundamental aspect of hospitality, but one that may lose sight of the more quotidian aspects of hospitality (Bulley 2015) as well as of hospitality’s affective dimensions and materialities (Zembylas 2019). In educational contexts, these affective and material dimensions can be constituted as part of the ethics of hospitality that supports the teacher/learner encounter (Ruitenberg 2018). Combining these views of hospitality as embodied, affective, ground in both conversation and materialities are essential to a view of education as a practice of disarmament (Ingold 2018), an openness to being vulnerable that echoes Derrida’s (2000) aporias: the contradictory but yet equally valid notions of unconditional hospitality and reciprocity.

A disposition towards unconditional hospitality, and the leap of faith this requires for the letting go of the need for full control, is essential to academic hospitality, to the academic ‘conversations’ that are at the same time metaphors of hospitality and practices of hospitality. We acknowledge this may sound idealistic, however, we believe it is not utopian. These conversations require a rethinking of an academia that is increasingly based on metrics, markets and on individual performance, one where scholars are encouraged to compete with each other for ever shrinking resources. They require that academics are ‘open to’ and ‘open towards’ others, to engage with and co-construct ideas and arguments, and also include affective dimensions and rituals of care and concern for others. This is how we would like academia to be.

Equally, the concept of academic hospitality requires a radical re-reading through and from indigenous and displaced understandings of academic hospitality. Reading online academic hospitality through the Gaza strip and with the Gaza strip is a beginning for our work of decreation, deconstruction and even decolonising of the conceptual apparatus surrounding both the academy and hospitality, in a move towards what Nymanjoh has termed ‘convivial scholarship’ (Nyamnjoh 2019):

[…] a convivial scholarship that dwells less on zero-sum games of absolute winners and losers, encourages a disposition of incompleteness and humility through the reality of the ubiquity of debt and indebtedness, and finds strength in themes of interconnections, interdependences, compositeness, and incompleteness […].

For a range of reasons, academic hospitality and educational practices are increasingly becoming more reliant on technological tools. Whether because of political and military enforced isolation, as is the case of the Gaza Strip that was discussed here, or because of the isolation imposed by lockdowns to stop the Covid-19 pandemic, which is the case as we write, online academic work is imposing itself on academics as a necessity and an opportunity.

Through the analysis of data coming from an online research project which generated moments of virtual commensality (Imperiale 2018) and virtual gifts, we have highlighted the contradictory dimensions of virtual academic hospitality, which can generate joyous feelings of presence and closeness while, at the same time, painfully foregrounding absences and unbridgeable distances. The online space mimics relationships in close proximity while subverting them, leaving us to grapple with the resulting feeling of uncertainty and ambiguity, asking (but not answering) whether, and to what extent, we can establish human relations and connections in the sterile space of the online.

While it is too early and too soon in a change that will take time and reflection (and academic conversations) to disentangle, we recognise the great potential for political work that the transgression of boundaries between human and technology offers. As Donna Haraway (2006: 26) notes

[…] taking responsibility for the social relations of science and technology means refusing an antiscience metaphysics, a demonology of technology, and so means embracing the skilful task of reconstructing the boundaries of daily life, in partial connection with others, in communication with all of our parts. It is not just that science and technology are possible means of great human satisfaction, as well as a matrix of complex dominations. Cyborg imagery can suggest a way out of the maze of dualisms in which we have explained our bodies and our tools to ourselves.

Perhaps online academic hospitality is one step closer to Derrida’s unconditional hospitality, the letting in of people your senses cannot fully apprehend while, at the same time, engaging in the transformative practice of leading out, in the true spirit of *ex-ducere* (Ingold 2018). Undoubtedly, and importantly, it makes possible the stretching towards—and engaging with—communities that are constructed through affinity (Haraway 2006), despite physical barriers and across borders.

Perhaps, too, online academic hospitality is a poetic act, a moment, like the image on the beach of a name; something which enacts *manaakitanga*, not ‘entertanment’ or ‘good will’, but care offered digitally, for relationships of conviviality that move beyond the dualisms of bodies and technological tools while still acknowledging our longing for physical presence.

**If you say my name**

Gifts are in the feet.

They bring your voice to my ear.

Tears are in the hair

Your hand helps them

Rise up through my breath.

War wounds abound

And strength is here

Abundantly

But If you say my name

The world of death

Where I stand on a shoreline

With the drowned

Where I kneel in the smoking

Rubble with the bombed.

If you say my name

Where I shake, barefoot, on the shards of glass

Scattered across the bedroom.

Where I listen in to the words

which wound with accusation,

disappointment,

blame and anxiety.

If you say my name

When the letter comes,

and the key turns and the decision falls

and she is taken away.

If you say my name

As you said her name

In the garden, after the battle,

after death was done

and after war wounds

were wrapped in balm and shroud.

If you say my name.

Gifts are in the feet.

If you say may name.

Tears are in the hair.

War wounds abundantly.

If you say my name.

Say my name.

(Poem by Alison Phipps)

## Footnotes

1 We use ‘Global South’ not to refer to a geographical area, but as a geopolitical construct as used in Santos (2018).
